# Supplementary material for: Pan-cancer profiling of tumor-infiltrating natural killer cells through transcriptional reference mapping
Source: Nat Immunol. 2024 Jul 2;25(8):1445–59. doi: 10.1038/s41590-024-01884-z (PMC11291284; doi:10.1038/s41590-024-01884-z)
Supplement: Supplementary file 1 — Supplementary Fig. 1 and Tables 1–5 including references. [file 41590_2024_1884_MOESM1_ESM.pdf]

nature immunology

Supplementary information https://doi.org/10.1038/s41590-024-01884-z

Pan-cancer profiling of tumor-infiltrating natural killer cells through transcriptional reference mapping

In the format provided by the authors and unedited

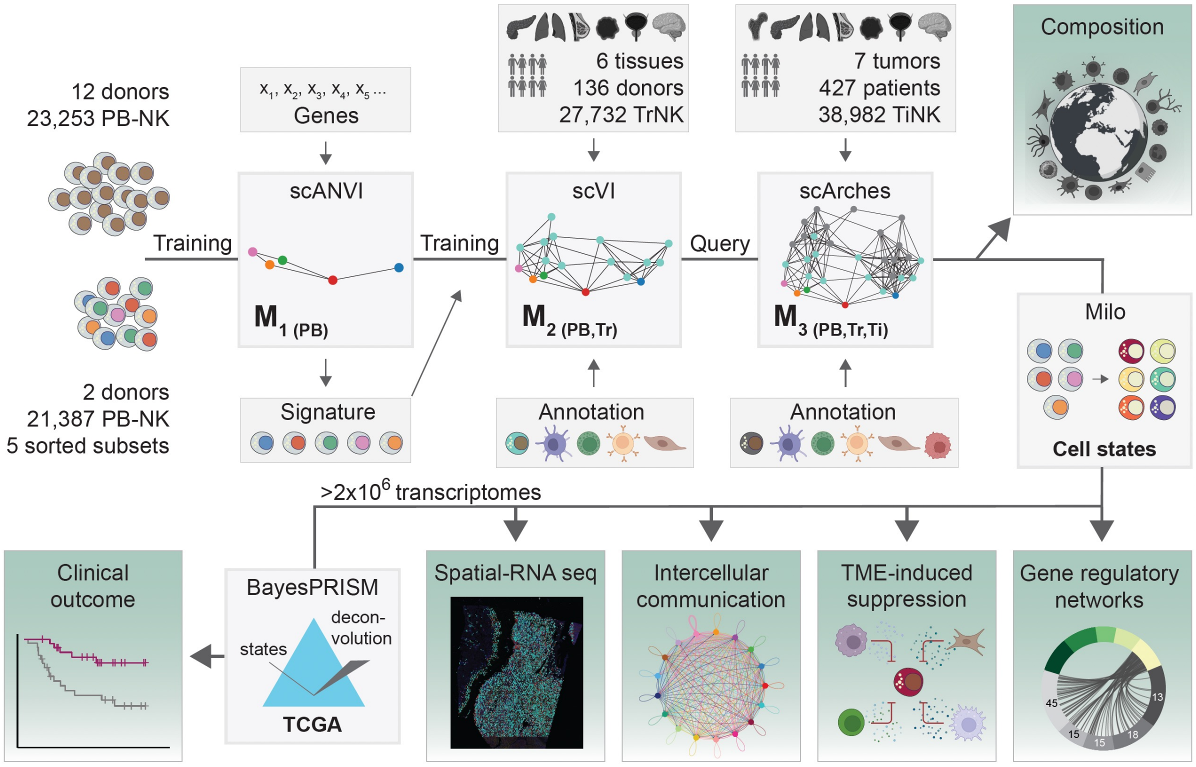

Supplementary Fig. 1: Graphical abstract.
Visualization of analysis workflow. The figure was partly generated using Biorender.

Supplementary tables

Supplementary table 1

Data containing NK cells from peripheral blood.

Dataset	Number of donors	Platform	Comment	Citation
Bulk PB NK cells	5	10x	Lab A in figure 1A	This manuscript
Sorted PB NK cells and bulk PB NK cells	2	10x	Lab B in figure 1A	This manuscript
Bulk PB NK cells	3	10x	Lab C in figure 1A	Crinier et al.[1]
Bulk PB NK cells	2	10x	Lab D ini figure 1A	Yang et al.[2]

Supplementary table 2

Data containing NK cells from normal tissue.

Dataset	Tissue type	# patients	Platform	Comment	Citation
Bulk tissue	Lung	7	10x	Tumor-adjacent normal lung samples, three from same patients as supplementary table 3	Lambrechts et al.[3]
Bulk tissue	Lung	4	10x	Tumor-adjacent normal lung samples, three from same patients as supplementary table 3	Chan et al.[4]
Bulk tissue	Lung	10	10x	Tumor-adjacent normal lung samples, same patients as supplementary table 3	Bischoff et al.[5]
Bulk tissue	Lung	7	10x	Tumor-adjacent normal lung samples, same patients as supplementary table 3	Goveia et al.[6]
Bulk tissue	Lung	11	10x	Tumor-adjacent normal lung samples, same patients as supplementary table 3	Kim et al.[7]
Bulk tissue	Lung	5	10x	Tumor-adjacent normal lung samples, same patients as supplementary table 3	He et al.[8]
Bulk tissue	Lung	42	10x	Tumor-adjacent normal lung samples, same patients as supplementary table 3	Leader et al.[9]
Bulk tissue	Pancreas	11	10x	3 cases of non-pancreatic tumor patients (e.g. bile duct tumors or duodenal tumors) and 8 cases of non-malignant pancreatic tumor patients (e.g. pancreatic cyst)	Peng et al.[10]
Bulk tissue	Pancreas	3	10x	adjacent/normal	Steele et al.[11]
Bulk tissue	Pancreas	3	10x	adjacent/normal	Chen et al.[12]
Bulk tissue	Prostate	10	10x		Tuong et al.[13]
Bulk tissue	Prostate	4	10x		Heidegger et al.[14]
Bulk tissue	Breast	10	10x	GEO: GSE161529	Bhat et al.[15]
Bulk tissue	Brain	4	10x		Siletti et al.[16]
Bulk tissue	Skin	3	10x	GEO: GSE169147	Damskey et al.[17]
Bulk tissue	Skin	6	10x	GEO: GSE193304	He et al.
Bulk tissue	Skin	3	10x	GEO: GSE160536	Mirizio et al.[18]
Bulk tissue	Skin	4	10x	GEO: GSE173205	Rindler et al.
Bulk tissue	Skin	4	10x	GEO: GSE138669	Xue et al.[19]

Supplementary table 3

Data containing NK cells from tumor tissue.

Dataset	Tumor type	# patients	Platform	Comment	Accession	Citation
Bulk TME	Lung (NSCLC)	8	10x		ArrayExpress: E-MTAB-6149 (5 patients) and E-MTAB-6653 (3 patients)	Lambrechts et al.[3]
Bulk TME	Lung (NSCLC)	7	inDrop		GEO: GSE127465	Zilionis et al.[20]
Bulk TME	Lung (NSCLC)	16	10x	HTAN		Chan et al.[4]
Bulk TME	Lung (NSCLC)	10	10x		DOI: 10.24433/CO.0121060.v1	Bischoff et al.[5]
Bulk TME	Lung (NSCLC)	49	10x	scRNA-seq and CITE-seq	GEO: GSE154826	Leader et al.[9]
Bulk TME	Lung (NSCLC)	5	10x		GSA: CRA001963	He et al.[8]
Bulk TME	Lung (NSCLC)	11	10x		SRA: PRJNA634159	Chen et al.[21]
Bulk TME	Lung (NSCLC)	8	10x		ArrayExpress: E-MTAB-6308	Goveia et al.[6]
Bulk TME	Lung (NSCLC)	11	10x		GEO: GSE131907	Kim et al.[7]
Bulk TME	Lung (NSCLC)	42	10x		GEO: GSE148071	Wu et al.[22]
Bulk TME	Pancreas (PDAC)	24	10x		GSA: CRA001160	Peng et al.[10]
Bulk TME	Pancreas (PDAC)	10	10x		GEO: GSE154778	Lin et al.[23]
Bulk TME	Pancreas (PDAC)	16	10x		GEO: GSE155698	Steele et al.[11]
Bulk TME	Pancreas (PDAC)	6	10x		GEO: GSE212966	Chen et al.[12]
Bulk TME	Melanoma	3	10x	Skin	GEO:GSE215120	Zhang et al.[24]
Bulk TME	Melanoma	8	10x	Skin		Smalley et al.[25]
Bulk TME	Glioblastoma (GBM)	4	10x		GEO: GSE162631	Xie et al.[26]
Bulk TME	Glioblastoma (GBM)	7	10x		Broad Insitute: SCP503	Richards et al.[27]
Bulk TME	Glioblastoma (GBM)	16	10x		GEO: GSE182109	Abdelfattah et al.[28]
Bulk TME	Glioblastoma (GBM)	7	10x		OSF: osf.io/4q32e/	Ravi et al.[29]
Bulk TME	Glioblastoma (GBM)	9	10x		GEO: GSE131928	Neftel et al.[30]
Bulk TME	Glioblastoma (GBM)	3	10x		GEO: GSE138794	Wang et al.[31]
Bulk TME	Glioblastoma (GBM)	3	10x		GEO: GSE139448	Wang et al.[32]

Dataset	Tumor type	# patients	Platform	Comment	Accession	Citation
Bulk TME	Glioblastoma (GBM)	5	10x		Synapse: syn22257780	Johnson et al.[33]
Bulk TME	Glioblastoma (GBM)	5	10x		GEO: GSE163108	Mathewson et al.[34]
Bulk TME	Glioblastoma (GBM)	13	10x		GEO: GSE163120	Pombo et al.[35]
Bulk TME	Glioblastoma (GBM)	35	10x		GEO: GSE154795	Lee et al.[36]
Bulk TME	Glioblastoma (GBM)	10	10x		GEO: GSE173278	LeBlanc et al.[37]
Bulk TME	Breast cancer	26	10x		GEO: GSE176078	Wu et al.[38]
Bulk TME	Breast cancer	34	10x		GEO: GSE161529	Pal et al.[39]
Bulk TME	Breast cancer	1	GEXSCOPE		GEO: GSE158399	Xu et al.[40]
Bulk TME	Breast cancer	5	GEXSCOPE		GEO: GSE180286	Xu et al.[41]
Bulk TME	Breast cancer	31	10x	treatment-naive pre-treatment	biokey. lambrechtslab.org	Bassez et al.[42]
Bulk TME	Prostate cancer	4	10x		GEO: GSE193337	Heidegger et al.[14]
Bulk TME	Prostate cancer	13	10x		GEO: GSE141445	Chen et al.[43]
Bulk TME	Prostate cancer	10	10x			Tuong et al.[13]
Bulk TME	Sarcoma	11	10x	Osteosarcoma	GEO: GSE152048	Zhou et al.[44]
Bulk TME	Sarcoma	6	10x	Osteosarcoma	GEO: GSE162454	Liu et al.[45]
Bulk TME	Sarcoma	4	10x	Osteosarcoma (TIL)	GEO: GSE198896	Cillo et al.

Supplementary table 4

Overview of number of cells from the various sources, including number of lymphoid cells and number of NK cells.

Dataset	Total cells in dataset	Lymphoid cell	NK cells
Peripheral blood			46303
Lung tumor	738416	344691	21608
Breast tumor	445376	147061	5130
Glioblastoma	782101	96838	5741
Pancreas tumor	124380	32614	1182
Sarcoma	150851	16328	1688
Prostate	66890	13131	549
Melanoma	28834	10754	3084
Lung normal	334072	119277	25137
Breast normal	46314	12125	646
Pancreas normal	38996	12584	673
Prostate normal	20717	6029	288
Brain normal		3236	718
Skin normal	137683	12112	270
——-	———	———	———
Total	2176214	826780	113017

Supplementary table 5

Overview of the spatial transcriptomics data sets used

Dataset	Staining Method	Link
Melanoma	IF	https://www.10xgenomics.com/datasets/human-melanoma-if-stained-ffpe-2-standard
Lung tumor	H&E	https://www.10xgenomics.com/datasets/human-lung-cancer-ffpe-2-standard
Glioblastoma	IF	https://www.10xgenomics.com/datasets/gene-and-protein-expression-library-of-human-glioblastoma-cytassist-ffpe-2-standard

References

1. Crinier, A. *et al.* High-Dimensional Single-Cell Analysis Identifies Organ-Specific Signatures and Conserved NK Cell Subsets in Humans and Mice. *Immunity* **49**, 971–986.e5 (2018).
2. Yang, C. *et al.* Heterogeneity of human bone marrow and blood natural killer cells defined by single-cell transcriptome. *Nat Commun* **10**, (2019).
3. Lambrechts, D. *et al.* Phenotype molding of stromal cells in the lung tumor microenvironment. *Nat Med* **24**, 1277–1289 (2018).
4. Chan, J. M. *et al.* Signatures of plasticity, metastasis, and immunosuppression in an atlas of human small cell lung cancer. *Cancer Cell* (2021) doi:10.1016/j.ccell.2021.09.008.
5. Bischoff, P. *et al.* Single-cell RNA sequencing reveals distinct tumor microenvironmental patterns in lung adenocarcinoma. *Oncogene* **40**, 6748–6758 (2021).
6. Goveia, J. *et al.* An Integrated Gene Expression Landscape Profiling Approach to Identify Lung Tumor Endothelial Cell Heterogeneity and Angiogenic Candidates. *Cancer Cell* **37**, 21–36.e13 (2020).
7. Kim, N. *et al.* Single-cell RNA sequencing demonstrates the molecular and cellular reprogramming of metastatic lung adenocarcinoma. *Nat Commun* **11**, 2285 (2020).
8. He, D. *et al.* Single-cell RNA sequencing reveals heterogeneous tumor and immune cell populations in early-stage lung adenocarcinomas harboring EGFR mutations. *Oncogene* **40**, 355–368 (2021).
9. Leader, A. M. *et al.* Single-cell analysis of human non-small cell lung cancer lesions refines tumor classification and patient stratification. *Cancer Cell* **39**, 1594–1609.e12 (2021).
10. Peng, J. *et al.* Single-cell RNA-seq highlights intra-tumoral heterogeneity and malignant progression in pancreatic ductal adenocarcinoma. *Cell Res* **29**, 725–738 (2019).
11. Steele, N. G. *et al.* Multimodal mapping of the tumor and peripheral blood immune landscape in human pancreatic cancer. *Nat Cancer* **1**, 1097–1112 (2020).
12. Chen, K. GEO: GSE212966. https://www.ncbi.nlm.nih.gov/geo/query/acc.cgi?acc=GSE212966.
13. Tuong, Z. K. *et al.* Resolving the immune landscape of human prostate at a single-cell level in health and cancer. *Cell Rep* **37**, 110132 (2021).
14. Heidegger, I. *et al.* Comprehensive characterization of the prostate tumor microenvironment identifies CXCR4/CXCL12 crosstalk as a novel antiangiogenic therapeutic target in prostate cancer. *Mol Cancer* **21**, 132 (2022).
15. Bhat-Nakshatri, P. *et al.* A single-cell atlas of the healthy breast tissues reveals clinically relevant clusters of breast epithelial cells. *Cell Rep Med* **2**, 100219 (2021).
16. Siletti, K. *et al.* Transcriptomic diversity of cell types across the adult human brain. 2022.10.12.511898 https://www.biorxiv.org/content/10.1101/2022.10.12.511898v1 (2022) doi:10.1101/2022.10.12.511898.
17. Damsky, W. *et al.* Inhibition of type 1 immunity with tofacitinib is associated with marked improvement in longstanding sarcoidosis. *Nat Commun* **13**, 3140 (2022).
18. Mirizio, E. *et al.* Single-cell transcriptome conservation in a comparative analysis of fresh and cryopreserved human skin tissue: Pilot in localized scleroderma. *Arthritis Res Ther* **22**, 263 (2020).
19. Xue, D. *et al.* Expansion of Fcγ Receptor IIIa-Positive Macrophages, Ficolin 1-Positive Monocyte-Derived Dendritic Cells, and Plasmacytoid Dendritic Cells Associated With Severe Skin Disease in Systemic Sclerosis. *Arthritis Rheumatol* **74**, 329–341 (2022).
20. Zilionis, R. *et al.* Single-Cell Transcriptomics of Human and Mouse Lung Cancers Reveals Conserved Myeloid Populations across Individuals and Species. *Immunity* **50**, 1317–1334.e10 (2019).
21. Chen, J. *et al.* Single-cell transcriptome and antigen-immunoglobin analysis reveals the diversity of B cells in non-small cell lung cancer. *Genome Biol* **21**, 1–21 (2020).
22. Wu, F. *et al.* Single-cell profiling of tumor heterogeneity and the microenvironment in advanced non-small cell lung cancer. *Nat Commun* **12**, 2540 (2021).

23. Lin, W. *et al.* Single-cell transcriptome analysis of tumor and stromal compartments of pancreatic ductal adenocarcinoma primary tumors and metastatic lesions. *Genome Med* **12**, 1–14 (2020).

24. Zhang, C. *et al.* A single-cell analysis reveals tumor heterogeneity and immune environment of acral melanoma. *Nat Commun* **13**, 7250 (2022).

25. Smalley, I. *et al.* Single-Cell Characterization of the Immune Microenvironment of Melanoma Brain and Leptomeningeal Metastases. *Clin Cancer Res* **27**, 4109–4125 (2021).

26. Xie, Y. *et al.* Key molecular alterations in endothelial cells in human glioblastoma uncovered through single-cell RNA sequencing. *JCI Insight* **6**, e150861.

27. Richards, L. M. *et al.* Gradient of Developmental and Injury Response transcriptional states defines functional vulnerabilities underpinning glioblastoma heterogeneity. *Nat Cancer* **2**, 157–173 (2021).

28. Abdelfattah, N. *et al.* Single-cell analysis of human glioma and immune cells identifies S100A4 as an immunotherapy target. *Nat Commun* **13**, 767 (2022).

29. Ravi, V. M. *et al.* T-cell dysfunction in the glioblastoma microenvironment is mediated by myeloid cells releasing interleukin-10. *Nat Commun* **13**, 925 (2022).

30. Neftel, C. *et al.* An Integrative Model of Cellular States, Plasticity, and Genetics for Glioblastoma. *Cell* **178**, 835–849.e21 (2019).

31. Wang, L. *et al.* The Phenotypes of Proliferating Glioblastoma Cells Reside on a Single Axis of Variation. *Cancer Discov* **9**, 1708–1719 (2019).

32. Wang, R. *et al.* Adult Human Glioblastomas Harbor Radial Glia-like Cells. *Stem Cell Reports* **14**, 338–350 (2020).

33. Johnson, K. C. *et al.* Single-cell multimodal glioma analyses identify epigenetic regulators of cellular plasticity and environmental stress response. *Nat Genet* **53**, 1456–1468 (2021).

34. Mathewson, N. D. *et al.* Inhibitory CD161 receptor identified in glioma-infiltrating T cells by single-cell analysis. *Cell* **184**, 1281–1298.e26 (2021).

35. Pombo Antunes, A. R. *et al.* Single-cell profiling of myeloid cells in glioblastoma across species and disease stage reveals macrophage competition and specialization. *Nat Neurosci* **24**, 595–610 (2021).

36. Lee, A. H. *et al.* Neoadjuvant PD-1 blockade induces T cell and cDC1 activation but fails to overcome the immunosuppressive tumor associated macrophages in recurrent glioblastoma. *Nat Commun* **12**, 6938 (2021).

37. LeBlanc, V. G. *et al.* Single-cell landscapes of primary glioblastomas and matched explants and cell lines show variable retention of inter- and intratumor heterogeneity. *Cancer Cell* **40**, 379–392.e9 (2022).

38. Wu, S. Z. *et al.* A single-cell and spatially resolved atlas of human breast cancers. *Nature genetics* **53**, 1334 (2021).

39. Pal, B. *et al.* A single-cell RNA expression atlas of normal, preneoplastic and tumorigenic states in the human breast. *EMBO J* **40**, e107333 (2021).

40. Xu, K. *et al.* Integrative analyses of scRNA-seq and scATAC-seq reveal CXCL14 as a key regulator of lymph node metastasis in breast cancer. *Hum Mol Genet* **30**, 370–380 (2021).

41. Xu, K. *et al.* Single-cell RNA sequencing reveals cell heterogeneity and transcriptome profile of breast cancer lymph node metastasis. *Oncogenesis* **10**, 66 (2021).

42. Bassez, A. *et al.* A single-cell map of intratumoral changes during anti-PD1 treatment of patients with breast cancer. *Nat Med* **27**, 820–832 (2021).

43. Chen, S. *et al.* Single-cell analysis reveals transcriptomic remodellings in distinct cell types that contribute to human prostate cancer progression. *Nat Cell Biol* **23**, 87–98 (2021).

44. Zhou, Y. *et al.* Single-cell RNA landscape of intratumoral heterogeneity and immunosuppressive microenvironment in advanced osteosarcoma. *Nat Commun* **11**, 6322 (2020).

45. Liu, Y. *et al.* Single-Cell Transcriptomics Reveals the Complexity of the Tumor Microenvironment of Treatment-Naive Osteosarcoma. *Front Oncol* **11**, 709210 (2021).
