# Supplementary material for: Pan-cancer profiling of tumor-infiltrating natural killer cells through transcriptional reference mapping
Source: Nat Immunol. 2024 Jul 2;25(8):1445–59. doi: 10.1038/s41590-024-01884-z (PMC11291284; doi:10.1038/s41590-024-01884-z)
Supplement: Supplementary file 2 — Reporting Summary [file 41590_2024_1884_MOESM2_ESM.pdf]

nature portfolio

Corresponding author(s): Aline Pfefferle, Karl-Johan Malmberg

Last updated by author(s): May 13, 2024

Reporting Summary

Nature Portfolio wishes to improve the reproducibility of the work that we publish. This form provides structure for consistency and transparency in reporting. For further information on Nature Portfolio policies, see our Editorial Policies and the Editorial Policy Checklist.

Statistics

For all statistical analyses, confirm that the following items are present in the figure legend, table legend, main text, or Methods section.

n/a	Confirmed	
☐	☒	The exact sample size (*n*) for each experimental group/condition, given as a discrete number and unit of measurement
☐	☒	A statement on whether measurements were taken from distinct samples or whether the same sample was measured repeatedly
☐	☒	The statistical test(s) used AND whether they are one- or two-sided<br>*Only common tests should be described solely by name; describe more complex techniques in the Methods section.*
☐	☒	A description of all covariates tested
☐	☒	A description of any assumptions or corrections, such as tests of normality and adjustment for multiple comparisons
☐	☒	A full description of the statistical parameters including central tendency (e.g. means) or other basic estimates (e.g. regression coefficient) AND variation (e.g. standard deviation) or associated estimates of uncertainty (e.g. confidence intervals)
☐	☒	For null hypothesis testing, the test statistic (e.g. *F*, *t*, *r*) with confidence intervals, effect sizes, degrees of freedom and *P* value noted<br>*Give P values as exact values whenever suitable.*
☐	☒	For Bayesian analysis, information on the choice of priors and Markov chain Monte Carlo settings
☒	☐	For hierarchical and complex designs, identification of the appropriate level for tests and full reporting of outcomes
☐	☒	Estimates of effect sizes (e.g. Cohen's *d*, Pearson's *r*), indicating how they were calculated

Our web collection on statistics for biologists contains articles on many of the points above.

Software and code

Policy information about availability of computer code

Data collection	R/Bioconductor package TCGAbiolinks (v 2.25.3)
Data analysis	10x Genomics Cell Ranger (v 7.0.0), r-base (v 4.2.3), python (v 3.9.16), scvi (v 0.20), CellChat (v 1.6.1), survival (v 3.5-5), survminer (0.4.9), scanpy (v 1.9.3), scikit-learn (v 1.2.2), scvelo (v 0.2.5), palantir (v 1.2), numpy (v 1.23.5), pyscenic (v 0.12.1), phenograph (v 1.5.7), arboreto (v 0.1.6), anndata (v 0.9.1), GSEA (v 4.2.3), edgeR (v 3.40.2), miloR (v 1.7.1), BayesPrism (v 2.0), decontX (v 1.0.0), Tangram (v 1.0.4), CellTypist (v 1.6.2), SOLO (v 1.0)<br><br>Code from our own analysis have been made available on GitHub: https://github.com/hernet/transcriptional-map-nk

For manuscripts utilizing custom algorithms or software that are central to the research but not yet described in published literature, software must be made available to editors and reviewers. We strongly encourage code deposition in a community repository (e.g. GitHub). See the Nature Portfolio guidelines for submitting code & software for further information.

Data

Policy information about availability of data

All manuscripts must include a data availability statement. This statement should provide the following information, where applicable:

- Accession codes, unique identifiers, or web links for publicly available datasets
- A description of any restrictions on data availability
- For clinical datasets or third party data, please ensure that the statement adheres to our policy

The gene expression data generated for this paper is available at NCBI GEO with accession number GSE245690 and raw sequencing data is available at EGA with accession number EGAS50000000014. The details about the publicly available data included in the analysis are available in Supplemental tables S1, S2, S3 and S5. Processed data and models have also been made available on Zenodo (https://zenodo.org/doi/10.5281/zenodo.8434223) and as an online resource at http://nk-scrna.malmberglab.com/. For GSEA the Molecular Signature Database (v2023.2.Hs) available at https://www.gsea-msigdb.org/gsea/msigdb/ was used. Relevant gene sets for scoring were also retrieved from this database. Bulk RNA-seq data was downloaded from TCGA and TARGET. Curated survival data was downloaded from Xena.

Research involving human participants, their data, or biological material

Policy information about studies with human participants or human data. See also policy information about sex, gender (identity/presentation), and sexual orientation and race, ethnicity and racism.

Reporting on sex and gender	Donors are anonymous and sex/gender is not discussed.
Reporting on race, ethnicity, or other socially relevant groupings	Donors are anonymous and race/ethnicity/social groupings are not discussed.
Population characteristics	Donors are anonymous.
Recruitment	Peripheral mononuclear cells (PBMC) were isolated using density gradient centrifugation from anonymized healthy blood donors (Oslo University Hospital; Karolinska University Hospital) with informed consent.
Ethics oversight	The study was approved by the regional ethics committee in Norway (Regional etisk komité (REK): 2018/2482) and Sweden (Regionala etikprövningsnämnden i Stockholm: 2016/1415-32, Etikprövningsmyndigheten: 2020-05289).

Note that full information on the approval of the study protocol must also be provided in the manuscript.

Field-specific reporting

Please select the one below that is the best fit for your research. If you are not sure, read the appropriate sections before making your selection.

☒ Life sciences ☐ Behavioural & social sciences ☐ Ecological, evolutionary & environmental sciences

For a reference copy of the document with all sections, see nature.com/documents/nr-reporting-summary-flat.pdf

Life sciences study design

All studies must disclose on these points even when the disclosure is negative.

Sample size	No sample size calculation was performed. Instead the sample size was chosen based on availability of material and published datasets at the time of study.
Data exclusions	No data was excluded from the analysis. Filtering and quality control of sequencing datasets are describe in the method section.
Replication	Different datasets were utilized as replicates for statistical analysis. More than 3 biologically independent replicates were used for all in vitro experiments. Data in Fig 4 f-g and Extended Data Fig. 6c-e is from 23 independent experiments, Fig. 6h is from 9 independent experiments, Fig. 6i is from 6 independent experiments, Fig. 6j-k from one independent experiment, Extended Data Fig. 9b-d is from 2 independent experiments
Randomization	Randomization was not relevant to this study as it involves the analysis of pre-existing datasets where conditions already have been applied. The analysis is also descriptive in nature and does not involve an intervention and there is no experimental manipulation to test.
Blinding	Blinding was not relevant to this study. Data was collected from existing datasets and the analysis aims to identify patterns and describe the transcriptional landscape of NK cells using computational methods, and blinding is not relevant in this context.

Behavioural & social sciences study design

All studies must disclose on these points even when the disclosure is negative.

Study description	*Briefly describe the study type including whether data are quantitative, qualitative, or mixed-methods (e.g. qualitative cross-sectional, quantitative experimental, mixed-methods case study).*
Research sample	*State the research sample (e.g. Harvard university undergraduates, villagers in rural India) and provide relevant demographic information (e.g. age, sex) and indicate whether the sample is representative. Provide a rationale for the study sample chosen. For studies involving existing datasets, please describe the dataset and source.*
Sampling strategy	*Describe the sampling procedure (e.g. random, snowball, stratified, convenience). Describe the statistical methods that were used to predetermine sample size OR if no sample-size calculation was performed, describe how sample sizes were chosen and provide a rationale for why these sample sizes are sufficient. For qualitative data, please indicate whether data saturation was considered, and what criteria were used to decide that no further sampling was needed.*
Data collection	*Provide details about the data collection procedure, including the instruments or devices used to record the data (e.g. pen and paper, computer, eye tracker, video or audio equipment) whether anyone was present besides the participant(s) and the researcher, and whether the researcher was blind to experimental condition and/or the study hypothesis during data collection.*
Timing	*Indicate the start and stop dates of data collection. If there is a gap between collection periods, state the dates for each sample cohort.*
Data exclusions	*If no data were excluded from the analyses, state so OR if data were excluded, provide the exact number of exclusions and the rationale behind them, indicating whether exclusion criteria were pre-established.*
Non-participation	*State how many participants dropped out/declined participation and the reason(s) given OR provide response rate OR state that no participants dropped out/declined participation.*
Randomization	*If participants were not allocated into experimental groups, state so OR describe how participants were allocated to groups, and if allocation was not random, describe how covariates were controlled.*

Ecological, evolutionary & environmental sciences study design

All studies must disclose on these points even when the disclosure is negative.

Study description	*Briefly describe the study. For quantitative data include treatment factors and interactions, design structure (e.g. factorial, nested, hierarchical), nature and number of experimental units and replicates.*
Research sample	*Describe the research sample (e.g. a group of tagged Passer domesticus, all Stenocereus thurberi within Organ Pipe Cactus National Monument), and provide a rationale for the sample choice. When relevant, describe the organism taxa, source, sex, age range and any manipulations. State what population the sample is meant to represent when applicable. For studies involving existing datasets, describe the data and its source.*
Sampling strategy	*Note the sampling procedure. Describe the statistical methods that were used to predetermine sample size OR if no sample-size calculation was performed, describe how sample sizes were chosen and provide a rationale for why these sample sizes are sufficient.*
Data collection	*Describe the data collection procedure, including who recorded the data and how.*
Timing and spatial scale	*Indicate the start and stop dates of data collection, noting the frequency and periodicity of sampling and providing a rationale for these choices. If there is a gap between collection periods, state the dates for each sample cohort. Specify the spatial scale from which the data are taken*
Data exclusions	*If no data were excluded from the analyses, state so OR if data were excluded, describe the exclusions and the rationale behind them, indicating whether exclusion criteria were pre-established.*
Reproducibility	*Describe the measures taken to verify the reproducibility of experimental findings. For each experiment, note whether any attempts to repeat the experiment failed OR state that all attempts to repeat the experiment were successful.*
Randomization	*Describe how samples/organisms/participants were allocated into groups. If allocation was not random, describe how covariates were controlled. If this is not relevant to your study, explain why.*
Blinding	*Describe the extent of blinding used during data acquisition and analysis. If blinding was not possible, describe why OR explain why blinding was not relevant to your study.*

Did the study involve field work? ☐ Yes ☒ No

Reporting for specific materials, systems and methods

We require information from authors about some types of materials, experimental systems and methods used in many studies. Here, indicate whether each material, system or method listed is relevant to your study. If you are not sure if a list item applies to your research, read the appropriate section before selecting a response.

Materials & experimental systems

n/a	Involved in the study
☐	☒ Antibodies
☐	☒ Eukaryotic cell lines
☒	☐ Palaeontology and archaeology
☒	☐ Animals and other organisms
☒	☐ Clinical data
☒	☐ Dual use research of concern
☒	☐ Plants

Methods

n/a	Involved in the study
☒	☐ ChIP-seq
☐	☒ Flow cytometry
☒	☐ MRI-based neuroimaging

Antibodies

Antibodies used

Flow cytometric analysis was performed with the following antibodies: PE-Cy7 mouse anti-human Perforin (eBioscience, deltaG9, cat # 25-9994-42, 1/100), PE goat anti-human IgG Fc Secondary Antibody (eBioscience, cat # 12-4998-82, 1/200), V500 mouse anti-human CD3 (BD Biosciences, UCHT1, cat # 561417, 1/100), V500 mouse anti-human CD14 (BD Biosciences, MϕP9, cat # 561391, 1/100), V500 mouse anti-human CD19 (BD Biosciences, HIB19, cat # 561121, 1/100), Alexa Fluor 700 mouse anti-human Granzyme B (BD Biosciences, GB11, cat # 560213, 1/100), BUV395 mouse anti-human CD107a (BD Biosciences, H4A3, cat # 565113, 1/80), Pacific Blue mouse anti-human CD16 (BD Biosciences, 3G8, cat # 558122, 1/50), FITC mouse anti-human CD57 (BioLegend, HNK-1, cat # 359604, 1/50), Brilliant Violet 650 mouse anti-human CD38 (BioLegend, HB-7, cat # 356620, 1/50), Brilliant Violet 421 mouse anti-human CD158e1 (BioLegend, DX9, cat # 312714, 1/50), PE mouse anti-human HLA-E (BioLegend, 3D12, cat # 342604, 1/50), Brilliant Violet 650 mouse anti-human TNFa (BioLegend, Mab11, cat # 502938, 1/25), Brilliant Violet 785 mouse anti-human IFNg (BioLegend, 4S.B3, cat # 502542, 1/25), APC-Vio770 anti-human CD158a (Miltenyi Biotec, REA284, cat # 130-120-444, 1/10), PE-Vio770 mouse anti-human CD158a/h (Miltenyi Biotec, 11PB6, cat # 130-099-891, 1/10), PE anti-human CD159c (Miltenyi Biotec, REA205, cat # 130-119-776, 1/10), PE-Vio770 anti-human CD159a (Miltenyi Biotec, REA110, cat # 130-113-567, 1/10), VioBright FITC anti-human CD159a (Miltenyi Biotec, REA110, cat # 130-113-568, 1/100), PE-Cy5.5 mouse anti-human CD158b1/b2,j (Beckman Coulter, GL183, cat # A66900, 1/50), APC mouse anti-human CD159a (Beckman Coulter, Z199, cat # A60797, 1/25), ECD mouse anti-human CD56 (Beckman Coulter, N901, cat # A82943, 1/20), APC mouse anti-human CD158e1/e2 (Beckman Coulter, Z27.3.7, cat # A60795, 1/50), LIVE/DEAD Fixable Aqua Dead Stain kit, 405 nM (Life Technologies, cat # L34965, 1/200).

Validation

All antibodies used in this study were titrated on human PBMCs prior to usage. Validated staining was determined by FACS and compared to other validated antibodies.

Eukaryotic cell lines

Policy information about cell lines and Sex and Gender in Research

Cell line source(s)	The A549 cell line was purchased from ATCC.
Authentication	The cell line was fingerprinted prior to usage.
Mycoplasma contamination	The cells are mycoplasma tested regularly (Eurofins).
Commonly misidentified lines (See ICLAC register)	No commonly misidentified cell lines were used in this study.

Palaeontology and Archaeology

Specimen provenance	*Provide provenance information for specimens and describe permits that were obtained for the work (including the name of the issuing authority, the date of issue, and any identifying information). Permits should encompass collection and, where applicable, export.*
Specimen deposition	*Indicate where the specimens have been deposited to permit free access by other researchers.*
Dating methods	*If new dates are provided, describe how they were obtained (e.g. collection, storage, sample pretreatment and measurement), where they were obtained (i.e. lab name), the calibration program and the protocol for quality assurance OR state that no new dates are provided.*

☐ Tick this box to confirm that the raw and calibrated dates are available in the paper or in Supplementary Information.

Ethics oversight	*Identify the organization(s) that approved or provided guidance on the study protocol, OR state that no ethical approval or guidance was required and explain why not.*

Note that full information on the approval of the study protocol must also be provided in the manuscript.

Animals and other research organisms

Policy information about studies involving animals; ARRIVE guidelines recommended for reporting animal research, and Sex and Gender in Research

Laboratory animals	*For laboratory animals, report species, strain and age OR state that the study did not involve laboratory animals.*
Wild animals	*Provide details on animals observed in or captured in the field; report species and age where possible. Describe how animals were caught and transported and what happened to captive animals after the study (if killed, explain why and describe method; if released, say where and when) OR state that the study did not involve wild animals.*
Reporting on sex	*Indicate if findings apply to only one sex; describe whether sex was considered in study design, methods used for assigning sex. Provide data disaggregated for sex where this information has been collected in the source data as appropriate; provide overall numbers in this Reporting Summary. Please state if this information has not been collected. Report sex-based analyses where performed, justify reasons for lack of sex-based analysis.*
Field-collected samples	*For laboratory work with field-collected samples, describe all relevant parameters such as housing, maintenance, temperature, photoperiod and end-of-experiment protocol OR state that the study did not involve samples collected from the field.*
Ethics oversight	*Identify the organization(s) that approved or provided guidance on the study protocol, OR state that no ethical approval or guidance was required and explain why not.*

Note that full information on the approval of the study protocol must also be provided in the manuscript.

Clinical data

Policy information about clinical studies

All manuscripts should comply with the ICMJE guidelines for publication of clinical research and a completed CONSORT checklist must be included with all submissions.

Clinical trial registration	*Provide the trial registration number from ClinicalTrials.gov or an equivalent agency.*
Study protocol	*Note where the full trial protocol can be accessed OR if not available, explain why.*
Data collection	*Describe the settings and locales of data collection, noting the time periods of recruitment and data collection.*
Outcomes	*Describe how you pre-defined primary and secondary outcome measures and how you assessed these measures.*

Dual use research of concern

Policy information about dual use research of concern

Hazards

Could the accidental, deliberate or reckless misuse of agents or technologies generated in the work, or the application of information presented in the manuscript, pose a threat to:

No	Yes	
☐	☐	Public health
☐	☐	National security
☐	☐	Crops and/or livestock
☐	☐	Ecosystems
☐	☐	Any other significant area

Experiments of concern

Does the work involve any of these experiments of concern:

No	Yes	
☐	☐	Demonstrate how to render a vaccine ineffective
☐	☐	Confer resistance to therapeutically useful antibiotics or antiviral agents
☐	☐	Enhance the virulence of a pathogen or render a nonpathogen virulent
☐	☐	Increase transmissibility of a pathogen
☐	☐	Alter the host range of a pathogen
☐	☐	Enable evasion of diagnostic/detection modalities
☐	☐	Enable the weaponization of a biological agent or toxin
☐	☐	Any other potentially harmful combination of experiments and agents

Plants

Seed stocks	N/A
Novel plant genotypes	N/A
Authentication	N/A

ChIP-seq

Data deposition

☒ Confirm that both raw and final processed data have been deposited in a public database such as GEO.

☒ Confirm that you have deposited or provided access to graph files (e.g. BED files) for the called peaks.

Data access links<br>*May remain private before publication.*	Publicly available datasets were used. Relevant GEO accession numbers can be found in the supplemental tables.
Files in database submission	N/A
Genome browser session<br>(e.g. UCSC)	N/A

Methodology

Replicates	N/A
Sequencing depth	N/A
Antibodies	N/A
Peak calling parameters	N/A
Data quality	N/A
Software	N/A

Flow Cytometry

Plots

Confirm that:

☒ The axis labels state the marker and fluorochrome used (e.g. CD4-FITC).

☒ The axis scales are clearly visible. Include numbers along axes only for bottom left plot of group (a 'group' is an analysis of identical markers).

☒ All plots are contour plots with outliers or pseudocolor plots.

☒ A numerical value for number of cells or percentage (with statistics) is provided.

Methodology

Sample preparation	Peripheral mononuclear cells (PBMC) were isolated using density gradient centrifugation from anonymized healthy blood donors (Oslo University Hospital; Karolinska University Hospital) with informed consent (Norway: Regional etisk komité (REK): 2018/2482, Sweden: Regionala etikprövningsnämnden i Stockholm: 2016/1415-32, Etikprövningsmyndigheten: 2020-05289). PBMC were stained for surface antigens and viability in a 96 V-bottom plate, followed by fixation/permeabilization and intracellular staining at room temperature.
Instrument	Samples were acquired on an LSR-Fortessa equipped with a blue, red and violet laser or sorted using a FACSAriaII (Beckton Dickinson).
Software	Data was analyzed in FlowJo version 9 and 10 (TreeStar, Inc.).
Cell population abundance	All cell populations contained > 100 cells and 12,000 cells were sorted for each sample.
Gating strategy	Gating strategies are show in the supplemental figures. Restrictive gates were used to ensure clean sorted populations. Post-sort purity testing was performed. Single-color stains and fluorescence minus one (FMO) were used as controls to set PMT voltages.

☒ Tick this box to confirm that a figure exemplifying the gating strategy is provided in the Supplementary Information.

Magnetic resonance imaging

Experimental design

Design type	*Indicate task or resting state; event-related or block design.*
Design specifications	*Specify the number of blocks, trials or experimental units per session and/or subject, and specify the length of each trial or block (if trials are blocked) and interval between trials.*
Behavioral performance measures	*State number and/or type of variables recorded (e.g. correct button press, response time) and what statistics were used to establish that the subjects were performing the task as expected (e.g. mean, range, and/or standard deviation across subjects).*

Acquisition

Imaging type(s)	*Specify: functional, structural, diffusion, perfusion.*
Field strength	*Specify in Tesla*
Sequence & imaging parameters	*Specify the pulse sequence type (gradient echo, spin echo, etc.), imaging type (EPI, spiral, etc.), field of view, matrix size, slice thickness, orientation and TE/TR/flip angle.*
Area of acquisition	*State whether a whole brain scan was used OR define the area of acquisition, describing how the region was determined.*

Diffusion MRI ☐ Used ☐ Not used

Preprocessing

Preprocessing software	*Provide detail on software version and revision number and on specific parameters (model/functions, brain extraction, segmentation, smoothing kernel size, etc.).*
Normalization	*If data were normalized/standardized, describe the approach(es): specify linear or non-linear and define image types used for transformation OR indicate that data were not normalized and explain rationale for lack of normalization.*
Normalization template	*Describe the template used for normalization/transformation, specifying subject space or group standardized space (e.g. original Talairach, MNI305, ICBM152) OR indicate that the data were not normalized.*

Noise and artifact removal

Describe your procedure(s) for artifact and structured noise removal, specifying motion parameters, tissue signals and physiological signals (heart rate, respiration).

Volume censoring

Define your software and/or method and criteria for volume censoring, and state the extent of such censoring.

Statistical modeling & inference

Model type and settings

Specify type (mass univariate, multivariate, RSA, predictive, etc.) and describe essential details of the model at the first and second levels (e.g. fixed, random or mixed effects; drift or auto-correlation).

Effect(s) tested

Define precise effect in terms of the task or stimulus conditions instead of psychological concepts and indicate whether ANOVA or factorial designs were used.

Specify type of analysis: ☐ Whole brain ☐ ROI-based ☐ Both

Statistic type for inference

(See Eklund et al. 2016)

Specify voxel-wise or cluster-wise and report all relevant parameters for cluster-wise methods.

Correction

Describe the type of correction and how it is obtained for multiple comparisons (e.g. FWE, FDR, permutation or Monte Carlo).

Models & analysis

n/a	Involved in the study
☐	☐ Functional and/or effective connectivity
☐	☐ Graph analysis
☐	☐ Multivariate modeling or predictive analysis

Functional and/or effective connectivity

Report the measures of dependence used and the model details (e.g. Pearson correlation, partial correlation, mutual information).

Graph analysis

Report the dependent variable and connectivity measure, specifying weighted graph or binarized graph, subject- or group-level, and the global and/or node summaries used (e.g. clustering coefficient, efficiency, etc.).

Multivariate modeling and predictive analysis

Specify independent variables, features extraction and dimension reduction, model, training and evaluation metrics.
